# Common bean reaction to angular leaf spot comprises transcriptional modulation of genes in the ALS10.1 QTL

**DOI:** 10.3389/fpls.2015.00152

**Published:** 2015-03-12

**Authors:** Paula R. Oblessuc, Cleverson C. Matiolli, Alisson F. Chiorato, Luis E. A. Camargo, Luciana L. Benchimol-Reis, Maeli Melotto

**Affiliations:** ^1^Department of Plant Sciences, University of California, DavisDavis, CA, USA; ^2^Departamento de Genética e Evolução e Bioagentes, Instituto de Biologia, Universidade Estadual de CampinasCampinas, Brazil; ^3^Centro de Pesquisa e Desenvolvimento em Recursos Genéticos Vegetais, Instituto Agronômico de Campinas—IACCampinas, Brazil; ^4^Centro de Grãos e Fibras, Instituto Agronômico de Campinas—IACCampinas, Brazil; ^5^Departamento de Fitopatologia, Escola Superior de Agricultura Luiz de Queiroz, Universidade de São PauloPiracicaba, Brazil

**Keywords:** *Phaseolus vulgaris*, *Pseudocercospora griseola*, plant disease response, gene expression, resistance locus, susceptible locus

## Abstract

Genetic resistance of common bean (*Phaseolus vulgaris* L.) against angular leaf spot (ALS), caused by the fungus *Pseudocercospora griseola*, is conferred by quantitative trait loci (QTL). In this study, we determined the gene content of the major QTL ALS10.1 located at the end of chromosome Pv10, and identified those that are responsive to ALS infection in resistant (CAL 143) and susceptible (IAC-UNA) genotypes. Based on the current version of the common bean reference genome, the ALS10.1 core region contains 323 genes. Gene Ontology (GO) analysis of these coding sequences revealed the presence of genes involved in signal perception and transduction, programmed cell death (PCD), and defense responses. Two putative R gene clusters were found at ALS10.1 containing evolutionary related coding sequences. Among them, the Phvul.010G025700 was consistently up-regulated in the infected IAC-UNA suggesting its contribution to plant susceptibility to the fungus. We identified six other genes that were regulated during common bean response to *P. griseola*; three of them might be negative regulators of immunity as they showed opposite expression patterns during resistant and susceptible reactions at the initial phase of fungal infection. Taken together, these findings suggest that common bean reaction to *P. griseola* involves transcriptional modulation of defense genes in the ALS10.1 locus, contributing to resistance or susceptibility depending on the plant-pathogen interaction.

## Introduction

One of the main objectives of molecular genetics is to identify and characterize genes governing important traits. Reverse and forward genetics are powerful approaches to achieve this objective. For plant species with considerable genetics and genomic resources, such as *Arabidopsis thaliana* (L. Heyhn.) for example, reverse genetic has been shown to be an important tool for gene discovery and functional analysis. However, for species with little or inexistent genomic resources, forward genetics by positional cloning is an efficient alternative technology for gene discovery, especially those controlling traits of agronomic importance in plants (Li et al., 2012; Xia et al., 2012). *Phaseolus vulgaris* L. (common bean) is one of the latter, and despite its social and economic importance (Broughton et al., 2003; Gepts et al., 2008), common bean genomics has lagged behind other crops. Nonetheless, the first version of the bean genome was released in 2014 (Schmutz et al., 2014) providing a significant stepping-stone for advancements in the field.

Until now, genetic maps have been the main source for positional cloning of genes of interest (Gepts et al., 2008) and subsequent functional analyses. Resistance (R) loci with nucleotide binding—leucine rich repeat (NB-LRR) and/or kinase domains, associated with resistance against devastating diseases, such as anthracnose (Creusot et al., 1999; Melotto et al., 2004), bean common mosaic virus (Vallejos et al., 2006) and common bacterial blight (Shi et al., 2011), have been identified. For instance, an R gene cluster was located on chromosome Pv04 (David et al., 2009; Geffroy et al., 2009), with the discovery of surrounding genes that are differentially expressed under biotic and abiotic stresses (David et al., 2010). This bean chromosome also harbors resistance genes against several strains of *Colletotrichum lindemuthianum* and *Pseudocercospora griseola* (López et al., 2003; Oblessuc et al., 2012, 2014; Ferreira et al., 2013; Gonçalves-Vidigal et al., 2013).

The fungus *P. griseola* (Sacc.) Crous and Braun (Crous et al., 2006) is the causal agent of angular leaf spot (ALS), a common bean disease with one of the greatest impact in yield, leading to losses as high as 80% and found in more than 60 countries around the world (Stenglein et al., 2003; Miklas et al., 2006). The molecular and biochemical mechanisms involved in this pathosystem are not established yet. Little is known about the infection process, except for limited morphological information available for *P. griseola* spore germination and hypha penetration through the epidermis or stomata (Monda et al., 2001). The hemibiotrophic life cycle of this fungus comprises of intercellular hyphae growth in the plant leaf mesophyll during the biotrophic phase, and subsequent hyphae penetration of the host cell causing plasmolysis during the fungus necrotrophic phase on susceptible plant genotypes (Monda et al., 2001).

Current knowledge on the genetics of the *P. vulgaris*—*P. griseola* pathosystem is restricted to the identification of molecular markers linked to different resistance loci and allelism tests (Ferreira et al., 2000; Sietsche et al., 2000; López et al., 2003; Mahuku et al., 2009; Gonçalves-Vidigal et al., 2011). Until now, six resistance genes for ALS were identified (*Phg-1* to *Phg-6*), and allelism tests revealed that different bean genotypes have different number and allele types. For instance, one of the most important sources of resistance to ALS, the AND 277 genotype, carries the *Phg*-1^a^, *Phg-2^2^, Phg-3^2^*, and *Phg-4^2^* loci (Mahuku et al. 2004; Caixeta et al., 2005; Gonçalves-Vidigal et al., 2011). Dominant monogenic inheritance for resistance to ALS has also been reported for other resistance sources (Namayanja et al., 2006; Amaro et al., 2007; Silva et al., 2008; Gonçalves-Vidigal et al., 2013). Gene characterization or functional analyses, however, have never been conducted to understand the function of the genes present in these regions.

In addition to qualitative resistance genes, resistance to ALS was also assigned to quantitative trait loci (QTL) of major and minor effects on the phenotypic variation observed in segregating populations. Among the QTL controlling resistance to ALS, ALS10.1 is a major QTL that was mapped to linkage group Pv10 using the IAC-UNA × CAL 143 (UC) recombinant inbreed line (RIL) population (Oblessuc et al., 2012). The ALS10.1 genome core was defined by integrating information on common bean genomic and genetic data using the six molecular markers that span the ALS10.1 region of 5.3 Mb (Oblessuc et al., 2013).

The genetic background where the QTL are present influences the expression of resistance (López et al., 2003; Mahuku et al., 2009, 2011; Oblessuc et al., 2012). The bean line CAL 143, derived from AND 277, shows resistance to a great number of *P. griseola* isolates in the field (Aggarwal et al., 2004; Oblessuc et al., 2012), in addition to resistance against rust (*Uromyces appendiculatus*), leaf spot (*Alternaria alternata*), and anthracnose (*Colletotrichum lindemuthianum*) (Vieira et al., 2002), highlighting the importance of this line as a source of broad disease resistance. Thus, the resistance locus ALS10.1 from CAL 143 was selected for the discovery of disease responsive genes. Here, we provide evidences that the ALS10.1 locus is enriched for genes involved in signal perception and transduction that includes two putative R gene clusters containing evolutionarily related genes, and putative negative regulators of immunity. We also showed that these defense-related genes on ALS10.1 are transcriptional regulated according to the plant's reaction to fungal infection. This study provides a significant step for functional characterization of this major common bean QTL and enhances our current understanding of the biological mechanisms that lead to bean resistance against angular leaf spot.

## Material and methods

### Functional gene annotation

All the predicted genes in a 5.5-Mb genomic region (Chr10:3,500,000 … 9,000,000) between the ALS10.1 core border markers IAC61 and BM157 (Oblessuc et al., 2013) were identified using the common bean genome database Phytozome v1.0 (http://www.phytozome.net/). Although a QTL for ALS and anthracnose was mapped to the same region of the ALS10.1 using the DOR364 × G19833 (López et al., 2003), there is no information in the literature regarding the functionality of the ALS10.1 QTL in the G19833 bean reference genotype. The Gene Ontology (GO) enrichment was assessed using the common bean gene locus ID from Phytozome as input for the Singular Enrichment Analysis (SEA), publicly available at AgriGO (http://bioinfo.cau.edu.cn/agriGO; Du et al., 2010). The SEA parameters used were: *P. vulgaris* v1.0 as background reference, Fisher test with the Yecutieli (FDR under dependency) multi-test adjustment method (*p* < 0.05), and complete GO gene ontology type. Significant third and fourth child terms of the GO term hierarchy (Blake and Harris, 2008) were identified within the major categories; biological process, cellular component, and molecular function. Additionally, we identify the closest putative ortholog in Arabidopsis for each bean gene through the gene homology annotation tool available in Phytozome v1.0 (threshold *E*-value ≤ 1 × 10^−20^ and identity > 10%).

### Phylogenetic analysis

The TIR-NB-ARC common bean protein Phvul.010G025700.1 was used as query for BLASTP (threshold *E*-value ≤ 1 × 10^−20^) against the common bean proteome (Phytozome v1.0; http://www.phytozome.net/). The identified proteins and the Phvul.010G025700.1 sequences were aligned with CLUSTALW as part of the MEGA 6.06 software (Tamura et al., 2013). The domains TIR and NB-ARC of the predicted Phvul.010G025700.1 were identified by searching this protein sequence against the NCBI protein conserved domain database (CDD) (Marchler-Bauer et al., 2015). The proteins containing both conserved domains (TIR and NB-ARC) were used to obtain the phylogenetic tree with MEGA 6.06 using the neighbor-joining method with partial deletion (Tamura et al., 2013). Bootstrap support values were obtained over 1000 replications.

### Pathogenesis assay and gene expression analysis

Seeds of the common bean genotypes CAL 143 (ALS resistance source) and the IAC-UNA (ALS susceptibility source) were placed on germination paper (Germilab, PR, Brazil) in a growth chamber at 25°C with 12 h photoperiod for 3 days. Seedlings were transplanted to pots filled with Dystrophic Red Latosol soil, fertilized with NPK 04-14-08 (400 kg/ha). Fungus culturing, inoculum preparation, and inoculation were performed as previously described (Oblessuc et al., 2012). Inoculated plants of each genotype were maintained in the greenhouse for 17 days to verify disease symptoms. Mock-inoculated plants were used as control for gene expression analysis.

Leaf samples were collected 22, 40, and 64 h post inoculation (hpi). Leaf total RNA was isolated using the RNeasy plant mini kit (Qiagen, Valencia, CA), according to manufacturer's instructions, followed by a DNase treatment (DNAse I Amplification Grade, Invitrogen, Carlsbad, CA). Two micrograms of high quality total RNA, quantified using a NanoDrop spectrophotometer (Thermo 367 Scientific, Rockford, IL), was converted into cDNA using the SuperScript® III First-Strand Synthesis kit (Invitrogen, Carlsbad, CA).

Quantitative PCR (qPCR) was performed in a final volume reaction of 12.5 μl with 0.5 μl of cDNA (RT reaction above), 250 mM of each transcript-specific primer (Table S1), and the Platinum® SYBR® Green qPCR kit (Invitrogen, Carlsbad, CA) in an optical 96-well plate format that included a passive reference dye (ROX) according to the manufacturer's instructions. qPCR cycles consisted of one cycle of 50°C for 2 min, another cycle of 95°C for 10 min, 40 cycles of 95°C for 15 s and 60°C for 1 min, using the Applied Biosystems 7500 thermocycler (Applied Biosystems, Foster City, CA). To verify amplification of single, specific target cDNA, a melting-curve analysis was included according to the thermal profile as suggested by the manufacturer (Applied Biosystems). The amount of plant RNA in each sample was normalized using the reference gene *PvIDE* (Phvul.001G133200, Borges et al., 2012) and relative gene expression (mock- vs. pathogen-inoculated samples) was determined with the 2^−ΔΔC^_T_ method (Livak and Schmittgen, 2001). The genes analyzed were: Phvul.010G040900 (*ENHANCED DISEASE RESISTANCE3*-*like; PvEDR3-like*), Phvul.010G033800 (receptor-like kinase; RLK), Phvul.010G031900 (RLK), Phvul.010G053300 (histidine phosphatase), Phvul.010G052900 (unknown protein), Phvul.010G026300 (TIR-domain), and Phvul.010G025700 (TIR-NB-ARC). The expression level of each gene was assessed in technical triplicates and three biological replicates. Three fully expanded leaflets of the first leaf of a single plant were bulked to compose one biological replicate. Statistical analysis was performed using the two-tailed Student's *t*-test.

## Results

### The ALS10.1 QTL is enriched for genes involved in plant-pathogen interactions

**Figure 1:**
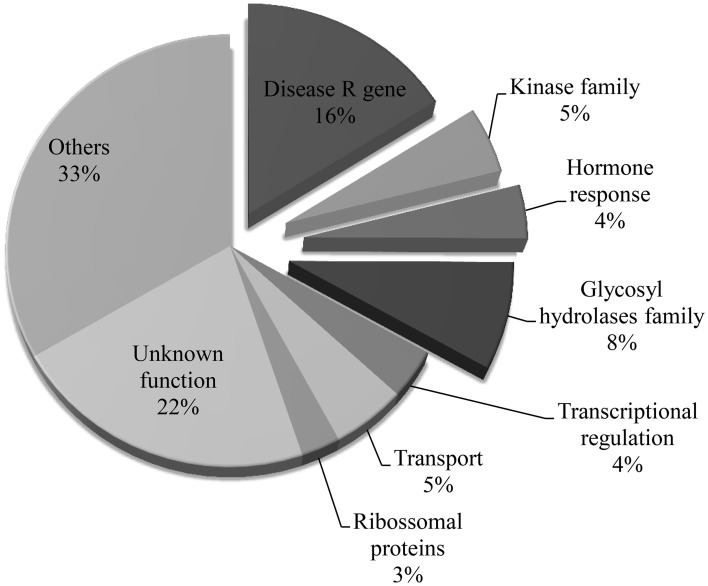
**Functional categories of the genes located in the ALS10.1 core**. Slices detached from the pie graph represent gene functions potentially related to immune responses. Percentage of genes in each category was calculated based on the total number of genes identified in the ALS10.1 core. The functional annotations were inferred based on the Phytozome v1.0 database.

Previously, we have defined the genomic boundaries of the ALS10.1 QTL using linked molecular markers (Oblessuc et al., 2013). To advance the current understanding of the functionality of ALS10.1 in common bean immunity, we first identified the predicted genes in this QTL region using the reference common bean genome sequence (Schmutz et al., 2014). A total of 323 genes were observed in that region and their functional annotation were inferred based on their putative domains and on the function of the most probable Arabidopsis ortholog (Table S2). A great number of genes functionally annotated as immune-related genes, such as TIR-NB-ARC domains coding genes, kinases, glycosyl hydrolases, and hormone responsive genes are present in the ALS10.1 core, which together represent 33% of all genes in that region (Figure 1). By comparison, approximately 1.1% of genes in the bean genome are annotated as involved in defense response (Table S3). Genes of unknown or other various functions were comprise 22% and 33% of this QTL, respectively (Figure 1).

**Figure 2:**
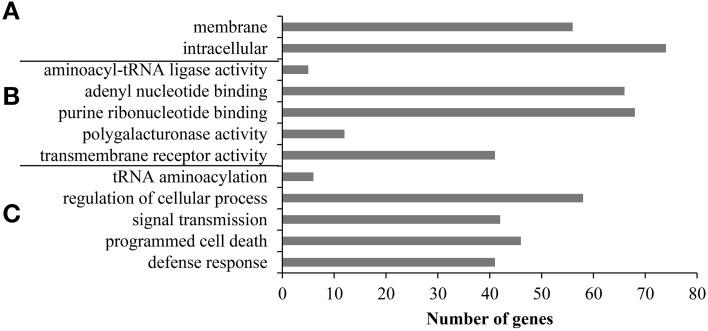
**Gene ontology (GO) enrichment analysis indicates that the ALS10.1 QTL is most likely involved in signal perception and transduction for cell defense**. Significant (FDR < 0.05) third and fourth child terms of the GO terms hierarchy for **(A)** cellular component, **(B)** molecular function, and **(C)** biological process categories. The x-axis indicates the number of genes observed within each GO term.

Next, we performed GO enrichment analysis to investigate whether the ALS10.1 locus contains genes involved in the same or convergent pathways and infer the overall function of ALS10.1. All 323 bean loci IDs were used as input for GO single enrichment analysis (SEA) revealing 49 GO categories significantly overrepresented (FDR ≤ 0.05) in the ALS10.1 locus (Table S3), and indicating a significant abundance of GO terms associated with signal perception and transduction, and immunity (Figure 2). Consistently, two GO categories within the cellular component domain, “membrane” (GO:0016020) and “intracellular” (GO:0005622), were overrepresented in the ALS10.1, with 56 and 74 genes, respectively. Both of these GO categories are child of the parental term “cell part” (GO:0044464), which includes 83 genes in the QTL (Table S3), indicating that some genes may code for protein with functions in the membrane and inside the cell. This finding is in agreement with the fact that all 41 genes in the molecular function GO term “transmembrane receptor activity” (GO:0004888) were also found in the “membrane” and “intracellular” categories (Table S3). In addition, the child terms of “nucleotide binding” (GO:0000166; 71 genes), “adenyl nucleotide binding” (GO:0032559; 66 genes) and “purine ribonucleotide binding” (GO:0032555; 68 genes), were also overrepresented in ALS10.1 (Figure 2), indicating an involvement of this locus in signal transduction. In fact, the “signal transmission” (GO:0023060) was a significant category within the biological process term containing 42 genes; 41 out of which are also associated with “transmembrane receptor activity.”

The biological process categories “programmed cell death” (GO:0012501) and “defense response” (GO:0006952) grouped 46 and 41 genes from the ALS10.1 locus, respectively (Figure 2). Interestingly, 37 genes were commonly placed in all “signal transmission,” “transmembrane receptor activity,” “defense response,” and “programmed cell death” GO categories (Table S3). The GO enrichment analysis supports the conclusion that the ALS10.1 genomic region, as a whole, carries important functions in plant-pathogen interactions.

**Figure 3:**
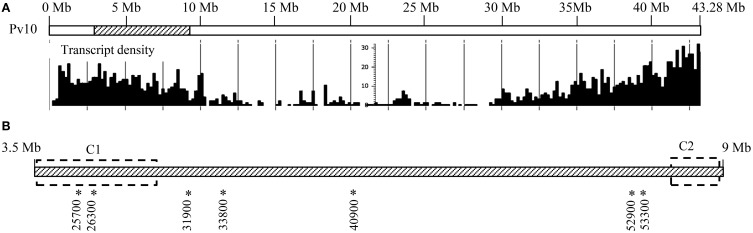
**ALS10.1 genomic location and disease-related genes position in the locus. (A)** Modified genome browser representation of the whole chromosome 10 (Pv10) where the position of the ALS10.1 locus is indicated by the striped region. The diagram below represents the transcript density of all Pv10 based on the common bean genome v.1.0 database (Schmutz et al., 2014; http://www.phytozome.net/). **(B)** Representation of the ALS10.1 genome region (Pv10:3,500,000 … 9,000,000). The dashed rectangles indicate the regions containing the two R gene clusters (C1 and C2). The double arrow represents the BAC clone P10A12 mapping position. Asterisks and numbers below the striped bar indicate the location and ID of genes analyzed by RT-qPCR.

Interestingly, 52 genes coding for TIR-NB-ARC, TIR, or NB-ARC domains (disease R gene category) were identified in the ALS10.1, representing 16.1% of all genes in this region (Figure 1). These genes are referred to as putative disease R genes based on the annotation of the closest Arabidopsis ortholog and on the fact that genes encoding an NB domain are commonly called R genes (Martin et al., 2003; van Ooijen et al., 2007); however, the actual molecular function of these common bean R genes remain elusive. We also found that these putative R genes are clustered in two opposite regions of the ALS10.1 core (Figure 3A); the first cluster spans 849 Kb at position 3,531,000 … 4,383,000 of Pv10 and contains 40 R genes; while the second cluster spans 311 Kb in the position 8,568,000 … 8,884,000 of Pv10 and harbors 12 R genes (Table S2 and Figure 3).

Thirty-nine of the 52 R genes (75%) have the highest similarity to a single Arabidopsis gene AT5G36930, annotated as TIR-NB-ARC domain-encoding gene (Table 1). Thirty-seven of them are located in the first R gene cluster. In this cluster, we also found the putative R genes: *PvSUMM2-like* (*SUPPRESSOR of MKK1 MKK2 2-like*), *PvTAO1-like* (*TARGET OF AvrB OPERATION 1*), and the AT5G48770-like that do not have a specific function established in Arabidopsis yet. The second R gene cluster contains the other two AT5G36930-like genes, and it is also enriched for genes with similarity to the TIR-NB-ARC-LRR domain-encoding gene AT5G17680 (8 out of the 12 R genes observed in this second cluster; 66.7%). We also found the putative *PvRPP5-like* (*RECOGNITION OF PERONOSPORA PARASITICA 5*), and one TIR-domain gene with no ortholog in Arabidopsis (*E*-value ≤ 1 × 10^−20^) on the second R gene cluster region of ALS10.1 (Table 1; Table S2).

**Table 1:** **Number of putative R genes located at the ALS10.1 QTL that share similarity to a single Arabidopsis gene and their functional annotation**.

**No. of bean transcripts**	**Arabidopsis ortholog**	**Arabidopsis protein annotation**	**GO category**
39	AT5G36930	TIR-NB-ARC	GO:0045087 innate immune response
			GO:0006915 apoptosis
			GO:0005524 ATP binding
			GO:0004888 transmembrane signaling receptor activity
			GO:0005515 protein binding
8	AT5G17680	TIR-NB-LRR	GO:0045087 innate immune response
			GO:0006915 apoptosis
			GO:0005524 ATP binding
			GO:0004888 transmembrane signaling receptor activity
			GO:0005515 protein binding
1	AT5G48770	TIR-NB-LRR	GO:0045087 innate immune response
			GO:0006915 apoptosis
			GO:0005524 ATP binding
			GO:0004888 transmembrane signaling receptor activity
			GO:0005515 protein binding
1	AT4G16950	RPP5	GO:0009817 defense response to fungus, incompatible interaction
			GO:0000166 nucleotide binding
1	AT5G44510	TAO1	GO:0042742 defense response to bacterium
1	AT1G12280	SUMM2	GO:0006952 defense response
			GO:0006915 apoptosis
			GO:0012505 endomembrane system
			GO:0005524 ATP binding
			GO:0005515 protein binding
1	–^*^	–	–

* This putative R genes did not share sequence similarity with any Arabidopsis gene.

### Putative R genes at ALS10.1 may be evolutionary related

Due to the great number of TIR-NB-ARC coding genes in the ALS10.1 and the fact that 37 R genes in cluster one (C1, Figure 3B) are similar to AT5G36930 and 8 R genes in cluster two (C2, Figure 3B) are similar to AT5G17680, we performed a phylogenetic analysis to infer the evolutionary relationship among these genes, as well as with other genes coding for TIR-NB-ARC domains in the common bean genome. The Phvul.010G025700.1 predicted protein contains the complete TIR-NB-ARC domains as the great majority (75%) of the R proteins of the ALS10.1 locus (Table 1 and Table S2) and it is regulated by fungal infection as explained below, we used this protein as query for BLASTP analysis (threshold *E*-value < 1 × 10^−20^) against the common bean proteome. We found 159 highly similar proteins in the bean proteome; however, only 108 of them contain both TIR (pfam01582) and NB-ARC (pfam00931) domains and were used for further protein analysis. Twenty-one of these proteins are translated from transcript splice variants, thus the phylogenetic tree was constructed with the query and the remainder 87 proteins predicted from the first splice variant of each gene (Figure S1).

**Figure 4:**
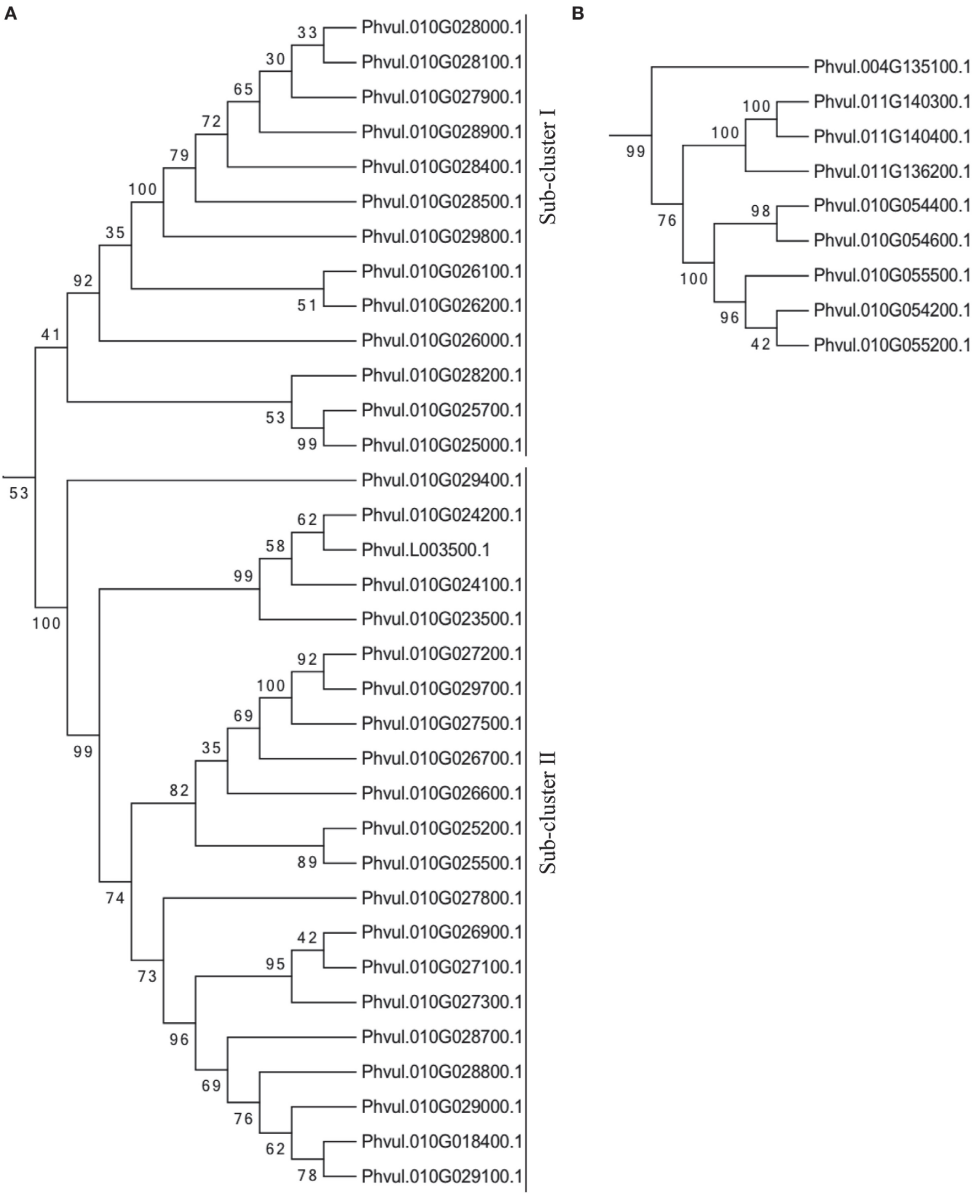
**The TIR-NB-ARC enconding genes in the ALS10.1 region are likely to be evolutionary related**. Phylogenetic analysis of predicted amino acid sequence based on Phytozome v1.0 database (http://www.phytozome.net) was performed with the Neighbor-joining method using the MEGA 6.06 software (Tamura et al., 2013). Bootstrap support values are provided adjacent to nodes. Clades contain predicted TIR-NB-ARC proteins from the first **(A)** and second **(B)** R gene clusters located at the ALS10.1 QTL region. (Refer to Figure S1 for the entire tree with the 88 TIR-NB-ARC proteins most similar to Phvul.010G025700.1 in the bean reference genome).

All TIR-NB-ARC proteins from the first R gene cluster at ALS10.1 (C1), with exception of Pvhul.010G025400.1 that contains an incomplete TIR domain, formed a unique clade along with two proteins that are just outside of the ALS0.1 region (Phvul.010G018400 and Phvul.010G023500) and the Phvul.L003500 located in the scaffold_40 of the bean genome reference (Figure 4A). This major clade can be divided into two sub-clusters, in which the sub-cluster I was formed with 13 proteins including the query protein Phvul.010G025700. The query protein is closely related to the proteins Phvul.010G025000.1 and Phvul.010G028200.1, indicating these three genes may be close paralogs (Figure 4A). Sub-cluster II contains 21 proteins including the ones outside the ALS10.1 locus on chromosome 10. These results indicate a complex evolution of these R genes on the ALS10.1 locus; and also suggest that the scaffold_40 could be part of the Pv10.

Five proteins from the second R gene cluster (C2) at the ALS10.1 region also formed a clade with three proteins from Pv11 and one from Pv04 (Figure 4B), suggesting that crossing-over may have occurred between these chromosomes and duplication may have happened on Pv10 resulting in these five related proteins. The other two proteins from the second R gene cluster annotated as containing TIR-NB-ARC domains, Phvul.010G054500 and Phvul.010G055100, were not used on the phylogeny analysis as they do not have a complete TIR domain.

### Genes in the ALS10.1 are differentially regulated upon pathogen infection

The genome and bioinformatics analyses described above and our previous genetic analysis of the ALS10.1 QTL (Oblessuc et al., 2013) support the notion that genes in this region work in concert to contribute for bean resistance against pathogens. Seeking further support for this hypothesis, we assessed whether resistant and susceptible phenotypes observed upon infection with the ALS fungal pathogen *P. griseola* were associated with transcriptional regulation of the genes in the ALS10.1 genomic region. A large number of possible biotic stress-responsive genes based on the genes annotation (Figure 1) and GO analysis (Figure 2) were found in the ALS10.1 core, however efficient primer sets with specific dissociation curve for RT-qPCR were only found for seven genes (Figure 3B) out of the 35 tested (data not shown), possibly due to the existence of extensive repetitive sequences that region. The time course for the gene expression analysis was chosen based on the developmental stages of *Colletotrichum higginsianum* and *C*. *graminicola* infection. Similar to *P. griseola, Colletotrichum* is a hemibiotrophic fungus that infects common bean with well-defined stages of infection (O'Connell et al., 2012). For instance, this fungus develops appressoria to penetrate the plant leaf around 22 hpi, colonizes the intracellular space during the biotrophic phase until around 40 hpi, and advances to the necrotrophic phase causing plasmolysis of the host cells after 60 hpi (O'Connell et al., 2012).

**Figure 5:**
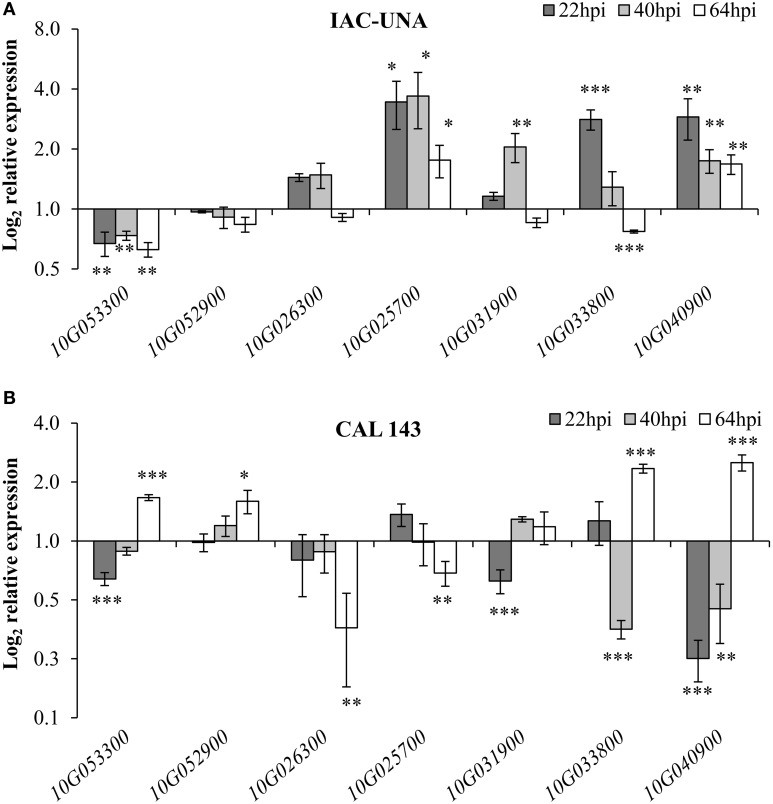
**ALS10.1 genes modulated during common bean interaction with *P. griseola***. Relative expression of the indicated genes (x-axis) in leaves of the common bean susceptible variety IAC-UNA **(A)** and in resistant line CAL 143 **(B)** sprayed with 1 x 10^4^ conidia/ml of *P. griseola*. Relative expression was calculated based on the gene expression in the mock-inoculated leaves (control) considered as 1. Data points are average of at least two biological replicates and six technical replicates. Asterisks above the standard error bars of all graphs indicate statistical significance calculated with Student's *t*-test (^*^ *p* < 0.05, ^**^ *p* < 0.01, ^***^ *p* < 0.001).

Interestingly, all seven genes tested showed differential expression during resistant (CAL 143) and/or susceptible (IAC-UNA) reactions to *P. griseola* infection as compared to the control mock-inoculated plants (Figure 5). The putative histidine phosphatase gene (Phvul.010G053300) was consistently repressed at all time points after inoculation of the susceptible variety IAC-UNA and a transient repression followed by induction in the resistant line CAL 143 (Figure 5). A gene of unknown function (Phvul.010G052900) and the TIR-domain encoding gene (Phvul.010G026300) were not responsive to the fungus infection in the susceptible variety IAC-UNA (Figure 5A). However, Phvul.010G052900 was induced and Phvul.010G026300 was repressed 64 hpi in the resistant line CAL 143 (Figure 5B) suggesting that these genes might have a role in immune response. On the other hand, the putative R gene Phvul.010G025700 was consistently induced at all time points during the susceptible response and only slightly down-regulated during the immune response only at 64 hpi (Figure 5), suggesting that this gene might have a role in disease susceptibility.

The two RLK-encoding genes (Phvul.010G031900 and Phvul.010G033800), the putative *ENHANCED DISEASE RESISTANCE3-like* (*PvEDR3-like*) (Phvul.010G040900) were up-regulated during the initial susceptible response of IAC-UNA (22–40 hpi) toward the ALS fungus, with a reduction in the expression levels at a later time of the infection (64 hpi) (Figure 5A). By contrast, these genes were repressed during the initial resistance response of sCAL 143, with up-regulation of transcription at later time of the infection (64 hpi), in which all but Phvul.010G031900 were significantly induced (Figure 5B). These results suggest that these two RLKs and the putative *PvEDR3-like* may play a role in a pathway that can be switched on or off according to resistant or susceptible reaction of the ALS pathogen (Figure 5).

Altogether, phylogeny and gene expression analyses suggest that resistance and susceptibility toward the ALS fungus is regulated, at least in part, at the transcriptional level, rather than presence or absence of specific genes or alleles in the ALS10.1 locus.

## Discussion

The identification of genes involved in common bean immunity is an important step to achieve durable genetic resistance in cultivated genotypes (Parlevliet, 1995; Johnson, 2000). One of the first steps to identify those genes is to study the genomic regions linked to resistance. The ALS10.1 is a major locus involved in ALS resistance (Oblessuc et al., 2012), a disease which causes great impact on common bean yield (Stenglein et al., 2003; Miklas et al., 2006). Here, we further characterized this genomic region by first identifying its genes based on the common bean genome annotation (Schmutz et al., 2014), followed by gene ontology, phylogeny, and transcriptional regulation analyses.

Gene Ontology (GO) enrichment analysis provides an overview of the response patterns implicated in a large set of genes. Therewith, we performed GO enrichment analysis for the 323 genes located at the ALS101.1 core region. Consistent with the genetic function on disease resistance previously assigned to this QTL, the GO terms overrepresented in the locus indicate enrichment for genes involved in signal perception and transduction, leading to activation of plant defenses. Indeed, 37 genes at ALS10.1 were predicted to be located at cell membrane also with an intracellular component, and transmembrane receptor and signal transmission activities, which may lead to defense response and programmed cell death (PCD), suggesting that ALS10.1 may play a role in the initial steps of the immune reaction to pathogens including signal perception at the membrane and subsequent transduction.

The R genes and the RLK are examples of known gene families that perceive and transduce signals through the cell membrane. Many R genes have been cloned in different species and the majority is predicted to encode intracellular multi-domain proteins with a C-terminal leucine-rich repeat (LRR) domain fused to a central nucleotide-binding (NB) domain (NB-LRR) (Martin et al., 2003; van Ooijen et al., 2007). The NB domain is part of a larger protein domain family named NB-ARC, because of its presence in APAF-1 (apoptotic protease-activating factor-1), R proteins, and CED-4 (*Caenorhabditis elegans* death-4 protein) (van der Biezen and Jones, 1998). NB-ARC proteins form a subclass of the STAND (Signal Transduction ATPases with Numerous Domains) super family that function simultaneously as sensors, switch and response factors, and are involved in a variety of processes, including immunity and apoptosis (van Ooijen et al., 2008). Among the probable defense related genes in the ALS10.1 locus, two clusters of putative R genes were identified. The Arabidopsis R gene AT5G36930 (TIR-NB-ARC) showed the highest similarity to the majority of the putative R genes in the ALS10.1 locus, and among them, the Phvul.010G025700 was the most similar (*E*-value = 2 × 10^−145^ and 33.3% identity). Indeed, both genes code for TIR-NB-ARC proteins, i.e., a N-terminal TIR domain followed by the NB-ARC domain.

Common bean R genes can be divided into TIR and non-TIR classes (Liu et al., 2012; Garzón et al., 2013), where members of the non-TIR class are found mostly in the chromosomes Pv04 and Pv11, and members of the TIR class are found mainly in Pv10 (Schmutz et al., 2014). Although TIR-NB-ARC proteins are found in all bean chromosomes, they are overrepresented on Pv10 and most of them (51.1%) are coded from genes in the ALS10.1 locus, highlighting the importance of this subfamily of R genes in the common bean response to *P. griseola*. Moreover, the high phylogenetic relationship among the R proteins from ALS10.1 indicates that the encoding genes underwent extensive duplication events, mainly in the first R gene cluster where all derived proteins formed a unique clade. Phylogeny analysis also indicated that cross-over may have occurred during the bean reference genotype (G19833) evolution between Pv10, Pv11, and Pv04 as putative TIR-NB-ARC paralogs from these chromosomes forms concise clades. Further molecular and biochemical analyses are needed to verify the function of these proteins in plant defense.

In Arabidopsis, the presence of either a coiled coil (CC) or TIR domain in the R protein typically determines whether an NB-LRR-mediated resistance response requires NDR1 (NON-RACE-SPECIFIC DISEASE RESISTANCE) or the EDS1 (ENHANCED DISEASE SUSCEPTIBILITY 1) signaling pathways, respectively (Aarts et al., 1998; Feys et al., 2005). Both pathway leads to SA-mediated PCD; however TIR-protein-induced PCD through EDS1 involves the autophagy machinery, while the CC-protein-mediated PCD is independent of autophagy (Hofius et al., 2009). This difference in response could be important for plant immunity against hemibiotrophic phytopathogens, such as the *P. griseola*. Plant defense responses needs to be coordinated according to the life phase of the pathogen. The biotrophic phase of the pathogen is more sensitive to the host PCD as it needs a live host cell to infect, while the host PCD would benefit the fungus at the necrotrophic phase.

Although the *P. griseola* hemibiotrophic life cycle during infection is not completely defined yet, one can infer its phases based on the life cycle of *Colletotrichum* sp. (O'Connell et al., 2012). Most likely, these two fungi have similar infection cycles as they both belong to the Ascomycota phylum with hemibiotrophic life cycle and infect the shoots of plants including common beans. Interestingly, we observed a correlation between gene expression level and the probable life phase of *P. griseola*, in which resistance may be achieved during the biotrophic phase of the fungus. The putative R genes (Phvul.010G026300 and Phvul.010G027500) were down-regulated only at 64 hpi in the resistant genotype, probably due to the fact that, at this late time point, the fungus infection had stop and the plant was restoring the physiological balance after infection (i.e., decrease defense responses). During the susceptible response to the ALS pathogen, Phvul.010G025700 was consistently induced, which lead us to hypothesize that the allele of IAC-UNA may be a target of effector-triggered susceptibility (Jones and Dangl, 2006). In this case, an effector may inhibit the resistance signal transduction leading to pathogen growth and disease. The fungus may also manage to induce the expression of this gene to promote its growth in the host and advance to the necrotrophic phase of the disease cycle. Further analysis of this bean gene will shed light on its molecular function in the common bean immune response.

Four genes had specific regulation direction (up- or down-regulation) depending on the time of fungus infection. During the probable fungus biotrophic phase, the two RLKs (Phvul.010G033800 and Phvul.010G031900) and the putative *PvEDR3-like* (Phvul.010G040900) were repressed in the resistance response of CAL 143 to *P. griseola*, whereas these genes were induced during the susceptible response of IAC-UNA. However, in the later timers of the resistant reaction, these genes were up-regulated reinforcing the hypothesis that after resistance have been achieved, the normal physiological balance of the cell needs to be re-established by discontinuing the, now unnecessary, immune response. Therefore, it is possible that Phvul.010G033800, Phvul.010G031900, and Phvul.010G040900 may act as negative regulator of immunity in common bean.

The Phvul.010G033800 and Phvul.010G031900 genes code for RLK proteins. Among the many sub-classes of kinases, the RLK play an important role in signal perception and transduction, including pathogen recognition (Gao et al., 2009). The Arabidopsis RLK AT1G34420, a putative ortholog of Phvul.010G033800, was shown to be involved in SA response in a negative regulation manner (Meier and Gehring, 2008), being induced in Arabidopsis' susceptible response to cabbage leaf curl virus (CaLCuV) infection (Ascencio-Ibáñez et al., 2008). These finding further support the possible involvement of the RLKs Phvul.010G033800 and Phvul.010G031900 in the repression of immune response during fungal infection of common bean.

The Phvul.010G040900 gene encodes for a putative PvEDR3-like protein. This annotation was based on the function of its closest ortholog in Arabidopsis, the *AtEDR3* gene that encodes for a dynamin-related protein 1E (Tang et al., 2006). Interestingly, the Arabidopsis loss-of-function mutant for this gene (*edr3*) has increased disease resistance to powdery mildew (*Golovinomyces cichoracearum*), in a SA-dependent manner, whereas *edr3* plants displays enhanced susceptibility to the necrotrophic fungal pathogen *Botrytis cinerea* (Tang et al., 2006). The repression of the *PvEDR3-like* during the initial phase of the *P. griseola* in the resistant bean genotype CAL 143 suggests that this gene may have similar function as *AtEDR3* in immunity. Therefore, it is possible that common bean resistance against *P. griseola* is achieved at the biotrophic developmental phase of the fungus, at least in part by down-regulating the expression of immunity repressors at the ALS10.1 locus during the biotrophich phase of the fungus life cycle.

Our functional analysis of the highly effective ALS10.1 QTL that controls pathogen infection of common bean, advances the current understanding of the *P. vulgaris*-*P. griseola* interaction. We have provided strong genetic, genomic, and phenotypic evidence that genes in this QTL are regulated by fungus infection, in addition to dissecting complex temporal patterns of gene regulation according to the outcome of the interaction (resistance or susceptibility). In the future, it would be interesting to determine whether ALS10.1 is also regulated by other fungal pathogens of beans as the CAL143 line is resistance to *Uromyces appendiculatus, Alternaria alternata*, and *C. lindemuthianum* (Vieira et al., 2002). As disease incidence has one of the most detrimental effects on crop yield, it becomes crucial to understand the molecular mechanisms controlling host-pathogen interactions to develop genetic resistant varieties and consequently yield improvement.

## Author contributions

Conceived and coordinated the project: LR, MM. Provided materials and facility access: AC, LR, MM. Performed experiments: PO, CM. Analyzed data: PO, CM, LC, MM. Wrote the manuscript: PO, LR, MM. All authors have read and approved the final version of the manuscript.

### Conflict of interest statement

The authors declare that the research was conducted in the absence of any commercial or financial relationships that could be construed as a potential conflict of interest.
