# Supplementary material for: Common bean reaction to angular leaf spot comprises transcriptional modulation of genes in the ALS10.1 QTL
Source: Front Plant Sci. 2015 Mar 12;6:152. doi: 10.3389/fpls.2015.00152 (PMC4357252; doi:10.3389/fpls.2015.00152)
Supplement: Supplementary file 1 [file Table1.PDF]

Supplementary Material

Common bean reaction to angular leaf spot comprises transcriptional modulation of genes in the ALS10.1 QTL

Paula Rodrigues Oblessuc[1,2,3], Cleverson Carlos Matiolli[2], Alisson F. Chioratto[4], Luis Eduardo Aranha Camargo[5], Luciana Lasry Benchimol-Reis[3], and Maeli Melotto[1]*

[1] Department of Plant Sciences, University of California, Davis, CA 95616, USA
[2] Departamento de Genética e Evolução e Bioagentes, Instituto de Biologia, Universidade Estadual de Campinas (UNICAMP), Campinas, SP 13083-970, Brazil
[3] Centro de Recursos Genéticos Vegetais, Instituto Agronômico (IAC), Campinas, SP 13020-432, Brazil
[4] Centro de Grãos e Fibras, Instituto Agronômico (IAC), Campinas, SP 13020-432, Brazil
[5] Departamento de Fitopatologia, ESALQ, Universidade de São Paulo, Piracicaba, SP 13418-900, Brazil

***Correspondence:**
Dr. Maeli Melotto
University of California, Davis
Department of Plant Sciences
One Shields Avenue
Davis, CA 95616, USA
E-mail: melotto@ucdavis.edu

1. Supplementary Figures and Tables

Table S1 | Primer sequences for the RT-qPCR analysis of selected genes in the ALS10.1 QTL. All primers were designed so that the annealing temperature was between 58°C and 60°C, and PCR fragments were between 80 and 130 bp in length. The letters F and R in front of the primer sequence indicate the directions of annealing as forward and reverse, respectively.

Table S2 | Predicted gene transcripts in the ALS10.1 core region (Chr10:3,500,000..9,000,000) and their putative Arabidopsis homologs, with functional annotation inferred based on the Phytozome (http://www.phytozome.net/) and TAIR (http://www.arabidopsis.org) databases, respectively. Gene loci in bold, underlined letters were included in the phylogenetic analysis of putative R genes.

Table S3 | Gene Ontology (GO) enrichment analysis of the common bean genes located at the ALS10.1 QTL. Common bean gene locus ID was used as input (query) for the GO enrichment analysis using the complete GO profile from *Phaseolus vulgaris* v1.0 as background (bg) available at AgriGO (http://bioinfo.cau.edu.cn/agriGO). Statistical significance was calculated with corrected fisher test (FDR < 0.05). P = Biological process; F = Molecular function; C = Cellular component.

FIGURE S1 | Phylogenetic tree of 88 TIR-NB-ARC proteins closest related to the predicted Phvul.010G025700 protein from ALS10.1. Phylogenetic analysis of predicted amino acid sequence based on Phytozome v1.0 database (http://www.phytozome.net) was performed with the Neighbor-joining method using the MEGA 6.06 software (Tamura et al., 2013). Bootstrap support values are provided adjacent to nodes. The proteins from ALS10.1 locus are separated into two clades corresponding to the two R gene clusters. The blue and red boxes contain the predicted proteins from the first and second R gene clusters, respectively.

1.1. Supplementary Tables

Table S1 | Primer sequences for the RT-qPCR analysis of selected genes in the ALS10.1 QTL. All primers were designed so that the annealing temperature was between 58°C and 60°C, and PCR fragments were between 80 and 130 bp in length. The letters F and R in front of the primer sequence indicate the directions of annealing as forward and reverse, respectively.

Putative gene function	Gene locus ID	Primer sequence (5’-3’)
PvIDE	Phvul.001G133200	F-GAGAGACTATGAGGTTGAAGC<br>R-CCATGAACTCGTACACTTAAAG
Receptor-like kinase (RLK)	Phvul.010G033800	F-ATTGGTCAAGTGGGTTTTGC<br>R-GCAAGCATCTGGTTTCTGAC
PvEDR3-like	Phvul.010G040900	F-TCCAGCTACTACCCCAAATG<br>R-GGATAGTGTTCCTGAGAGTG
Histidine phosphatase	Phvul.010G053300	F-GGTGGCGTTTTCTTCATTCC<br>R-AAATTGGGCGTCTGAGTTGG
Protein of unknown function	Phvul.010G052900	F-GTTGGGGAGTGATAAGGAAG<br>R-GTTCTGGTAATGCTTGTTGAG
Receptor-like kinase (RLK)	Phvul.010G031900	F-ATTGCCCTTTGGTGTGTGC<br>R-GCACCTCCACTTCAACAAC
TIR-domain	Phvul.010G026300	F-CGTCGCATGTTCGTAATCAG<br>R-TCATCGTTCAACCCTTGAGG
TIR-NB-ARC	Phvul.010G025700	F-ATCAACCCCTGAAAACATGG<br>R-ACACCCTGCCAATCTTCATC
